# Age-shifting in malaria incidence upon cessation of interventions: a simulation study

**DOI:** 10.1186/1475-2875-13-S1-O16

**Published:** 2014-09-22

**Authors:** Peter Pemberton-Ross, Katya Galactionova, Tom Smith, Melissa Penny

**Affiliations:** 1 Swiss Tropical and Public Health Institute, Basel, Switzerland; 2 University of Basel, Basel, Switzerland

## Background

Malaria disease shifts to older ages when interventions are applied only to the youngest children, and incidence will resurge in endemic settings when intervention coverage decreases. This has consequences for the deployment of long-lasting insecticide treated nets or infection blocking vaccines (e.g. pre-erythrocytic vaccines). These delay acquisition of immunity, with the result that previously protected individuals may experience pathology later in life due to undeveloped or waning immunity.

Here we use a mathematical model to investigate the long-term population impact and cost-effectiveness of new interventions and address the reasons rebounds are observed in models when interventions are not renewed and relate to observations from field trials.

## Materials and methods

The age and magnitude of age- and time-shifting of incidence is investigated using an ensemble of individual-based stochastic simulation models of *Plasmodium falciparum* dynamics, with varied assumptions about immunity decay, transmission heterogeneity, and access to treatment. The impact of transmission intensity, levels of access to malaria treatment and primary levels of intervention coverage and intervention profile are also considered. The main drivers of shifts in incidence are determined, examining model assumptions of age incidence, immunity, and initial exposure. The likely onset and duration of excess events in a treated cohort compared to a control is also examined for specific intervention profiles.

## Results

In the absence of new interventions, or the redistribution of current interventions, we find that the underlying absence or loss of immunity resulting from previous lack of exposure is a primary driver of shifting of disease burden to older age groups. The simulations suggest that with short half-life interventions (~1 year), excess cases in the intervened cohort may be seen as soon as 3-4 years after intervention cessation. An excess of severe disease occurs sooner than one of uncomplicated disease, and sooner in high transmission areas than in low transmission areas. Because of slow build-up of natural immunity, excess incidence may be distributed over a long period after intervention cessation, making it unlikely that this will be detectable in field studies.

## Conclusions

This has important consequences for the design of transient interventions, in terms of both duration and demographics of deployment. Effective interventions will lead to initial decreases in malaria cases in a population, but these can be lost - or worse, redistributed in time and magnified in number and impact, without sustained efforts to decrease transmission.

